# Light Chain Myeloma with Highly Atypical Plasma Cells and Extensive Auer Rod-Like Inclusions

**DOI:** 10.4274/tjh.galenos.2018.2018.0197

**Published:** 2019-02-07

**Authors:** Dietmar Enko, Gernot Kriegshäuser

**Affiliations:** 1 General Hospital Steyr, Institute of Clinical Chemistry and Laboratory Medicine, Steyr, Austria; 2 Medical University Graz, Clinical Institute of Medical and Chemical Laboratory Diagnostics, Graz, Austria

**Keywords:** Light chain myeloma, Plasma cells, Bone marrow aspirate

## To the Editor,

A 73-year-old woman with a history of chronic kidney disease presented with fever (39.8 °C), dyspnea, and fatigue. Complete blood count showed moderate normocytic anemia with hemoglobin of 10.0 g/dL (normal range: 12.0-16.0), mild leukocytosis of 10.8x10^9^/L (normal range: 4.0-9.0), and thrombocytopenia of 102x10^9^/L (normal range: 150-400). Serum protein electrophoresis showed mild hypogammaglobulinemia of 6.7 g/L (normal range: 7.0-16.0). Serum immunofixation electrophoresis demonstrated monoclonal κ-type light chains without heavy chain correlates (IgG, IgM, IgA, IgD, IgE). Moreover, a serum-free light chain assay measured a high κ-type light chain level of 2060.0 mg/L (normal range: 3.3-19.4) with a κ/λ ratio of 48.5 (normal range: 0.3-1.7).

**Figure 1:**
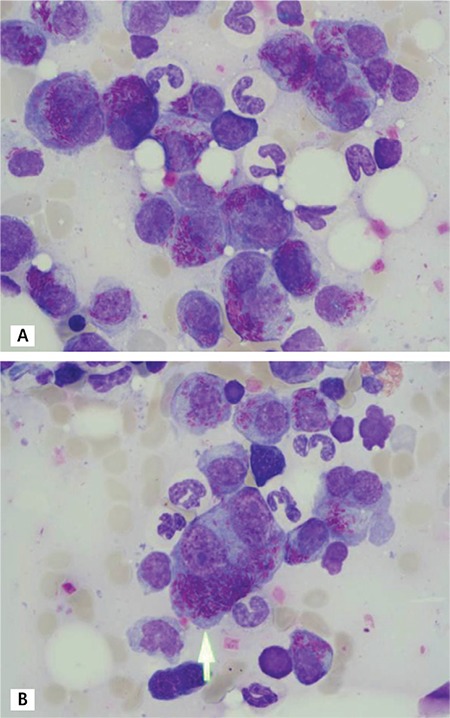
Bone marrow aspirate smear of a 73-year-old patient with κ-type light chain myeloma (A and B). The arrow marks a binuclear plasmablastic cell containing numerous Auer rod-like inclusions.

A bone marrow aspirate smear showed 40% plasma cells, many of which appeared as binuclear plasmablastic cells with nucleoli (“owl-eyed” plasma cells), bright cytoplasm, and bundles of numerous Auer rod-like cytoplasmic inclusions (Figures 1A and 1B). This unique morphology is remarkable. While the current literature describes Auer rod-like inclusions in single cases of different forms of myeloma [1,2,3,4,5], this is, to the best of our knowledge, the first report on the concomitant appearance with enlarged highly atypical “owl-eyed” plasma cells in a patient suffering from κ-type light chain myeloma. However, the prognostic value of this unusual plasma cell phenotype remains unclear.
